# Supplementary material for: Inverse-designed silicon nitride nanophotonics
Source: Nat Commun. 2026 May 28;17:6943. doi: 10.1038/s41467-026-73390-9 (PMC13389240; doi:10.1038/s41467-026-73390-9)
Supplement: Supplementary file 1 — Supplementary Information [file 41467_2026_73390_MOESM1_ESM.pdf]

Supplementary information for "Inverse-designed silicon nitride nanophotonics"

Toby Bi[1,2,†], Shuangyou Zhang[1,3,†], Egemen Bostan[4], Danxian Liu[4], Aditya Paul[4,5], Olga Ohletz[1], Irina Harder[1], Yaojing Zhang[6], Alekhya Ghosh[1,2], Abdullah Alabbadi[1,2], Masoud Kheyri[1,2], Tianyi Zeng[4], Jesse Lu[7], Kiyoul Yang[4,*], and Pascal Del'Haye[1,2,*]

[1]Max Planck Institute for the Science of Light, Staudtstraße 2, Erlangen, 91058, Germany.
[2]Department of Physics, Friedrich-Alexander Universität Erlangen-Nürnberg, Staudtstraße 7, Erlangen, 91058, Germany.
[3]Department of Electrical and Photonics Engineering, Technical University of Denmark, Kgs., Lyngby, 2800, Denmark.
[4]John A. Paulson School of Engineering and Applied Sciences, Harvard University, Cambridge, MA, 02138, USA.
[5]Department of Electrical Engineering and Computer Science, Massachusetts Institute of Technology, Cambridge, MA, 02138, USA.
[6]School of Science and Engineering, The Chinese University of Hong Kong (Shenzhen), Shenzhen, Guangdong, 518172, P.R. China.
[7]SPINS Photonics Inc, Hollister, CA, 95023, USA.
[†]These authors contributed equally: Toby Bi, Shuangyou Zhang
[*]Corresponding authors: pascal.delhaye@mpl.mpg.de, kiyoul@seas.harvard.edu

I. FABRICATION TOLERANCE

The optimisation routine assumes vertical sidewalls, no deviation from the designed thickness, minimum feature sizes achievable during fabrication and constant refractive index. In figures S1 and S2, the impact of fabrication tolerances, such as different thickness, refractive index, and sidewall angle are shown for the inverse-designed mirror and wavelength-division multiplexer shown in this work. In practice, a parametric sweep of the uniform scaling factor is performed to compensate for systematic fabrication deviations.

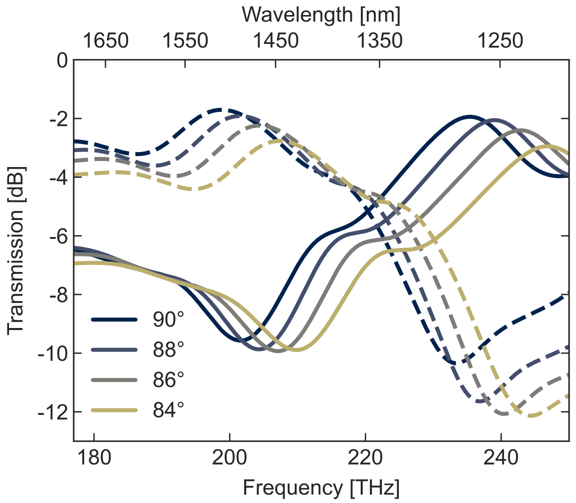

FIG. S1. Comparison of an inverse-designed C- and O-band wavelength-division multiplexer simulated with different sidewall angles.

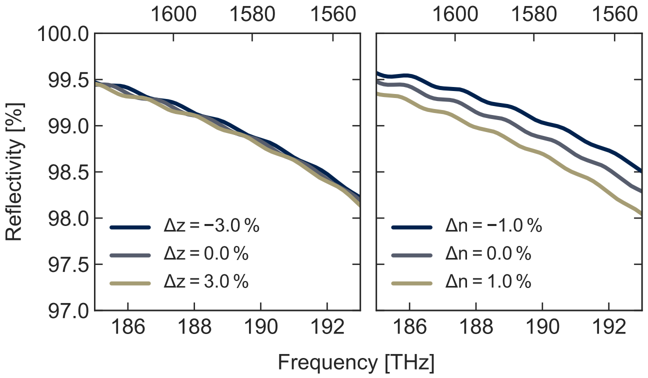

FIG. S2. Comparison of an inverse-designed mirror simulated with thickness (left panel) and refractive index (right panel) deviating from the ideal values of $z = 800\,\mathrm{nm}$ and $n = 2$.

II. IMPACT OF OPTIMISATION INITIAL CONDITIONS

Increasing the optimisation area and including additional closely spaced frequencies as objective functions seems to generally improve performance. This is demonstrated by optimising three WDMs with different initial conditions as shown in figure S3. Increasing the optimisation area from $5 \times 5\,\mu\mathrm{m}^2$ to an area of $7 \times 7\,\mu\mathrm{m}^2$ improved the insertion loss by 0.4 dB in both channels while having two closely spaced in frequency constraints for each channel, separated by 10 nm, instead of one led to 0.8 dB improvement in both channels.

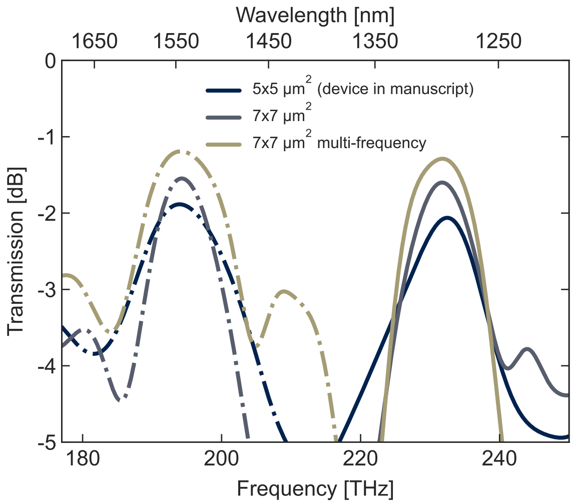

FIG. S3. Inverse-designed C- and O-band wavelength-division multiplexer optimised using different initial conditions.

III. INVERSE-DESIGNED FABRY-PÉROT CAVITIES

A. Integrated dispersion of FP cavities

Due to the addition of mirrors, the FP cavities offer greater flexibility in dispersion engineering than conventional ring resonators, where tuning is limited to the waveguide cross-section and/or ring radius. In this case here (shown in figure S4), the total cavity dispersion is strongly anomalous, $D_2/(2\pi) = 86.73\,\text{MHz}$, which supports higher power per comb-line in the operational band of the inverse-designed FP cavity. Note that the dispersion noted here is challenging to reach in a conventional ring geometry. In the figure S4, the integrated dispersion of a ring resonator is compared to the FP resonator with 144.6 GHz FSR, where the GVD is around 53 times stronger in the FP cavity than a ring resonator fabricated on the same chip. One can envisage the use of the strong GVD in the FP cavity to concentrate more power in the centre of the comb spectrum.

B. Comparison to other FP cavities and mirrors

The reflector design determines the microcavity finesse independently of the cavity FSR, and for a given finesse, the cavity Q-factor increases (decreases) as the cavity length increases (decreases). In the resonators shown in this work, the single mirror round-trip losses are 1.5 % while the cavity waveguide losses are 0.08 % per round-trip for an FSR of 144.6 GHz or 0.04 % per round-trip for an FSR of 307.6 GHz. If the cavity length is increased to 8.1 mm (equivalent to an FSR of 18.55 GHz[1]) the round-trip cavity waveguide losses are still only 0.68 %, therefore, the cavities shown here are mirror-loss limited. At

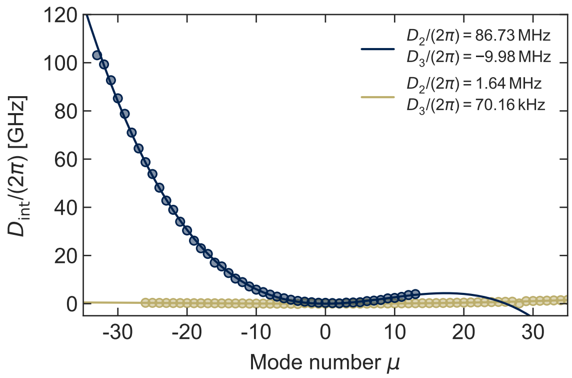

FIG. S4. Integrated dispersion of an inverse-designed FP cavity with FSR = 144.6 GHz (blue) compared to a ring resonator on the same chip (gold).

an FSR of 18.55 GHz, the Q-factor is 1.29 million, as shown in figure S6.

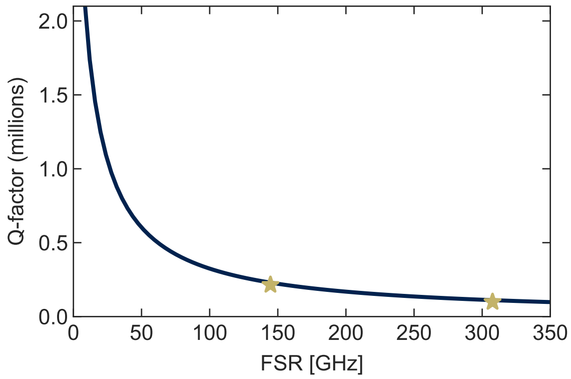

FIG. S5. Q-factor of an FP cavity as the FSR is varied, where the markers are the measured cavities in this work.

The mirrors in this work have a peak reflectivity of 98.5 % and a reflectivity above 95 % across a 4 THz bandwidth in a 2D footprint of $2.8 \times 11\,\mu\text{m}^2$. While the previously reported inverse-designed reflectors in silicon nitride[2] have a maximum reflectivity of $94.2 \pm 2.4\,\%$ in a similar 2D footprint of $4 \times 8\,\mu\text{m}^2$. This would result in a maximum Q-factor between 46,700 to 118,000 at 144.6 GHz, although a resonator is not reported in their work. In this case, we assume the best-case scenario that the in-cavity waveguide has the same propagation loss as our work. In practice, the single-mode waveguide reported in their work would have higher propagation losses compared to the multi-mode waveguide used here. As well as the higher demonstrated reflectivity, our reflectors are designed to function in transmission, further reducing the total required footprint for optical accessibility.

C. Simulated single mirror reflectivity

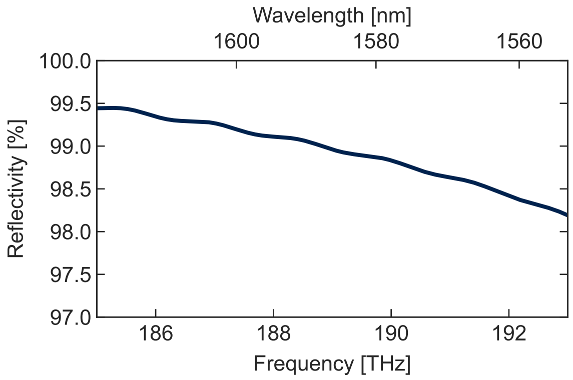

FIG. S6. Reflectivity of a single inverse-designed mirror for the quasi-TE_{00} in the same spectral window as shown in the main text.

D. Transmission of inverse-designed FP in the vicinity of 188 THz

As the cavity is shortened, the resonance linewidths increase. This increases spectral overlap between spatial mode families, making closely spaced resonances more difficult to distinguish. In the 307.6,GHz cavity, many resonances of the TE_{00} mode cannot be unambiguously distinguished from those of the TE_{10} mode. This is evident around 188,THz in figure S7, where the two mode families strongly overlap.

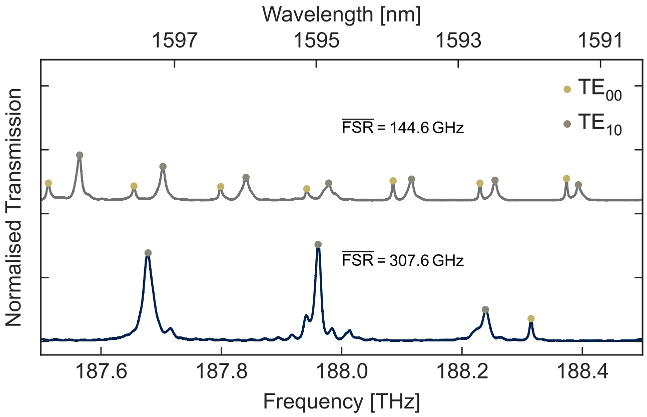

FIG. S7. Transmission of inverse-designed FP cavities in the vicinity of 188 THz.

SUPPLEMENTARY REFERENCES

[1] Wildi, T., Gaafar, M. A., Voumard, T., Ludwig, M. & Herr, T. Dissipative Kerr solitons in integrated Fabry–Perot microresonators. *Optica* **10**, 650–656 (2023).

[2] Pita, J., Nabki, F. & Ménard, M. Inverse-designed silicon nitride reflectors. *Optics Letters* **49**, 786–789 (2024).
