# Supplementary material for: Inverse-designed silicon nitride nanophotonics
Source: Nat Commun. 2026 May 28;17:6943. doi: 10.1038/s41467-026-73390-9 (PMC13389240; doi:10.1038/s41467-026-73390-9)
Supplement: Supplementary file 2 — Transparent Peer Review file [file 41467_2026_73390_MOESM2_ESM.pdf]

nature portfolio

Peer Review File

Inverse-Designed Silicon Nitride Nanophotonics

Corresponding Author: Dr Pascal Del'Haye

This file contains all reviewer reports in order by version, followed by all author rebuttals in order by version.

Version 0:

Reviewer comments:

Reviewer #2

(Remarks to the Author)
In this manuscript, the authors implemented inverse-designed photonics on the low-loss silicon nitride platform and demonstrated inverse-designed wavelength-division multiplexers, mode-division multiplexers, and F-P cavities formed by two reflectors, highlighting the potential of compact photonic components in silicon-nitride-based photonic systems.

However, it is not clear if this is a substantial improvement compared with others' work, especially the work presented in Ref 58 "Integrated silicon nitride devices via inverse design." I suggest that the authors provide more discussion comparing their work with others' and with previous research to better emphasize the advantages.

Overall, I believe the work is technically sound. The manuscript could be suitable for publication, provided that the authors thoroughly address my comments.

Main comments:

-In Fig. 3b, I am surprised to see that the measured crosstalk at ~188 THz is better than that in simulation. Do the authors have any insight into the reason for that?

-In Fig. 3d, there is a noticeable reduction in the crosstalk variation and insertion loss compared with that in Fig. 3b. Is this improvement a result of the larger film thickness and optimization area, or of a different optimization algorithm?

- If my understanding is correct, the only difference between the two F-P cavities in Fig. 4d is the length of the intracavity waveguide. I am curious why the transmission of the TE00 modes in the 307.6 GHz cavity is very different from that in the 144.6 GHz cavity. Did the authors use different reflector designs?

- The Q-factor of the F-P cavities is 0.21 million, which seems too low for practical applications. By comparison, a Q-factor of 4 million has been demonstrated in traditionally designed F-P cavities (Ref. 48). I believe the authors should discuss the limiting factors, possible ways to improve the Q-factor of the F-P cavities, and the maximum Q-factor that can be achieved in simulation.

Minor comments/suggestions:

- In Fig. 2b and 2d, it would be better to use 'Transmission (dB)' or 'Normalized transmission (dB)' as the label for the vertical axes, and the same applies to Fig. 3b and 3d.

-In the second paragraph of Section B, the authors state that "After around 200 iterations the optimized MDM achieves crosstalk below −21 dB and insertion loss of −4 dB…" The frequencies corresponding to these values should be specified, because both the crosstalk and insertion loss vary significantly with frequency in Fig. 3b.

Reviewer #3

(Remarks to the Author)
In this paper, the authors demonstrate a very high Q thick silicon nitride platform and use standard inverse design techniques to demonstrate a 2-mode multiplexer, 2-wavelength multiplexer, and mode selective reflector with unfortunately

mediocre insertion loss and overall performance.

The paper also comes at an unfortunate time as a cited preprint [58] demonstrates significantly larger devices with higher performance that perform more complex functions (4 wavelength multiplexer, 4 mode multiplexer, PBS). However, even if this preprint is not considered, the application of standard inverse design techniques to a silicon nitride platform is not unprecedented -
(1) Pita Ruiz, Julian L., Frederic Nabki, and Michaël Ménard. "Silicon nitride TM-pass polarizer using inverse design." Optics Express 31.23 (2023): 37892-37899.
(2) Ruiz, Julian L. Pita, Narges Dalvand, and Michaël Ménard. "Inverse-designed 90-degree silicon nitride bends for the C band." Journal of Lightwave Technology (2025).

While the high quality factor platform is impressive, as the authors note it is not the first demonstration of this. Moreover, the high quality factor becomes irrelevant when the insertion loss of devices is as high as it is here (exceeding 2dB). In the same vein, the design of a reflector that keeps the field away from the side walls seems completely superfluous as the intrinsic Q is 4 to 20 million and the Q of the demonstrated engineered resonators is 200,000.

Additional note:
The side wall angle appears to be relatively significant and may explain why some devices perform significantly worse than simulated even when fabrication tolerance is accounted for.

Although the work is presented well, I cannot recommend its publication in Nature Communications.

Version 1:

Reviewer comments:

Reviewer #2

(Remarks to the Author)
I have carefully reviewed the revised manuscript. The authors have comprehensively addressed all of my questions. Given the detailed author responses and careful revisions, I recommend the manuscript for publication in Nature Communications.

Reviewer #3

(Remarks to the Author)
While the authors have made improvements to the clarity of the writing of the paper, none of the major concerns of either review were actually addressed (low Q, high insertion loss) beyond explanations, and no new experiments have been done.

The paper is well written and sound, can and should be published, but due to the lack of performance of the devices shown and lack of innovation in method, I maintaing that it does not meet the standards for publication in Nature Communications.

Open Access This Peer Review File is licensed under a Creative Commons Attribution 4.0 International License, which permits use, sharing, adaptation, distribution and reproduction in any medium or format, as long as you give appropriate credit to the original author(s) and the source, provide a link to the Creative Commons license, and indicate if changes were made.
In cases where reviewers are anonymous, credit should be given to 'Anonymous Referee' and the source.
The images or other third party material in this Peer Review File are included in the article's Creative Commons license, unless indicated otherwise in a credit line to the material. If material is not included in the article's Creative Commons license and your intended use is not permitted by statutory regulation or exceeds the permitted use, you will need to obtain permission directly from the copyright holder.
To view a copy of this license, visit https://creativecommons.org/licenses/by/4.0/

Point-by-point response to the reviewers

Manuscript title: *Inverse-Designed Silicon Nitride Nanophotonics*
Manuscript ID: NCOMMS-25-47595

We thank the reviewers for their detailed insights and suggestions for the manuscript. All reviewer comments are address and given in black with our responses in blue. The changes in the manuscript and supplementary information are given in red.

Reviewer 1

In this manuscript, the authors implemented inverse-designed photonics on the low-loss silicon nitride platform and demonstrated inverse-designed wavelength-division multiplexers, mode-division multiplexers, and F-P cavities formed by two reflectors, highlighting the potential of compact photonic components in silicon-nitride-based photonic systems. However, it is not clear if this is a substantial improvement compared with others' work, especially the work presented in Ref 58 "Integrated silicon nitride devices via inverse design." I suggest that the authors provide more discussion comparing their work with others' and with previous research to better emphasize the advantages.

We thank the reviewer for the suggestion and agree that a clearer comparison to other work, particularly Ref. 58 ("Integrated silicon nitride devices via inverse design"), will help contextualise the contribution and advantages of our manuscript. We have therefore expanded the discussion comparing our demonstrated components to Ref. 58 below and to representative SiN implementations based on conventional design approaches. We would also like to emphasise that the work in Ref. 58 was done simultaneously and at the preprint stage at the same time like our work. According to the Nature publishing guidelines this should not be considered as impacting the novelty of our work[1].

For a more detailed comparison, Ref. 58 demonstrates inverse-designed SiN components (CWDM, multi-mode MDM, and a polarization beam splitter), with an emphasis on ultra-compact footprints and robustness (reporting up to 1200× footprint reduction with minimum feature sizes on the order of 160 nm) on 400 nm-thick films. In contrast, our work addresses a complementary regime and extends the demonstrated functionality in two main ways. (1) Beyond inverse-designed WDMs and MDMs, we additionally demonstrate microcavities formed by two inverse-designed reflectors, directly targeting applications in quantum nonlinear photonics. This provides an integrated resonant building block not addressed in Ref. 58, a thicker SiN layer is required to achieve favourable dispersion conditions. (2) We experimentally present an inverse-designed SiN nanophotonics library spanning wavelength, mode, and resonant functionalities on a low-loss SiN platform on up to 800 nm-thick films.

Second, to address the reviewer's request for context versus other approaches, we now explicitly compare against conventional SiN devices that typically rely on directional couplers or MMIs and therefore require substantially larger interaction lengths/footprints. For example, directional-coupler WDMs commonly require hundreds of microns coupling length: the WDM coupler shown in Fig. 4 of Ji et al. [1] uses a coupling length >700 µm (1480/1550 nm). A monolithically integrated SiN/Tm:Al_2O_3 platform reports a WDM directional coupler with length 570 µm (SiN width 0.9 µm, gap 1.5 µm)[2]. An Er:poly-Al_2O_3 amplifier integrated with Si photonics reports a WDM directional coupler with coupling length 330 µm (gap 0.9 µm)[3] and low-loss / low-crosstalk operation (0.5 dB insertion loss, −20 dB crosstalk). Finally, for visible-wavelength

[1] Quote from the Nature communications website: "Preprint archives do not compromise novelty. Papers that are published independently while your manuscript is under review or under revision at Nature Communications are also not considered to compromise novelty, even in cases where there is conceptual overlap."

MDM, "Mode-division multiplexing for visible photonic integrated circuits" reports a 200 nm-thick SiN platform and adiabatic-coupler section lengths $415 + 510 + 530$ m (total 1.455 mm), with crosstalk <-20 dB and insertion loss 3.55 dB for the TE-mode coupler[4].

Since the conventional and inverse-designed Fabry-Pérot (FP) cavities is addressed in a later comment, the comparison will be shown there.

Finally, Ref. 58 inverse-designed devices are optimised an order of magnitude more slowly than our devices. Ref. 58 reports that their optimisation takes between 15 minutes to 5.5 hours per iteration step, depending on the device, and up to 450 iterations per device optimisation. We thus infer that the CWDM, MDM, and PBS optimisation of Ref. 58 has taken between 16 days to 94 days, 2 days to 37 days, and 2 days to 17 days, respectively. The lower bound is optimistic as it assumes the shortest time for every iteration step while the pessimistic guess assumes the longest time for each step. Our optimisation methods take between 1 day and 3 days, using weaker and older hardware (4 Nvidia T4 GPUs, 16 GB VRAM each, released in 2018).

We believe these additions make it clear that (i) our manuscript is not a repetition of Ref. 58 but rather complements it by expanding the inverse-designed SiN device set to include reflector-based FP cavities and resonant functionality in a thicker platform, and (ii) compared to widely used SiN WDM/MDM implementations based on conventional couplers (often hundreds of µm to mm scale), inverse-design enables substantially more compact components while maintaining strong performance.

We have included a comparison to conventionally-designed components on comparative platforms in the **Introduction** of the main text.

Beyond benchmarking within our platform, it is useful to contrast these devices with more conventional SiN implementations, which typically rely on directional couplers or MMIs and therefore require substantially larger interaction lengths and footprints. Representative WDMs based on directional couplers often use coupling lengths of several hundred micrometers, and coarse WDM and mode-division multiplexers can occupy footprints up to the mm^2 scale. In contrast, the inverse-designed WDMs and MDMs presented here have footprints in the range of $5 \times 5\,\mu m^2$ to $8 \times 8\,\mu m^2$ (WDM) and $8 \times 8\,\mu m^2$ to $15 \times 10\,\mu m^2$ (MDM), corresponding to footprint reductions on the order of $\sim$50 to 300 times for the WDMs[1, 2, 3] and $\sim$500 times for the MDMs[4, 5] relative to representative coupler-, or resonator-based implementations, while operating on a significantly thicker, ultra-low-loss SiN platform.

In Fig. 3b, I am surprised to see that the measured crosstalk at 188 THz is better than that in simulation. Do the authors have any insight into the reason for that?

We agree with the reviewer that we found it surprising that the measured crosstalk is higher than initially designed. We do not believe that this is a limitation of the optimisation scheme but reflects that the simulation is an ideal design that does not account for fabrication tolerances which tend to have a greater effect for features in close proximity. Fabrication variations such as the thickness, material dispersion, surface roughness, proximity effects leading to over- or under-etching, and/or sidewall angle. This may actually improve device performance rather than hinder it especially for devices operating across a broad spectral window.

In the supplementary information, we have included simulations that show device performance changing with fabrication tolerances such as thickness, refractive index, dispersion, and sidewall angle. The figures below show the change in the inverse-designed mirror reflectivity where the thickness and refractive index are changed from the ideal designed value ($z = 800$ nm and $n = 2$) and the WDM performance as the sidewall angle deviates from 90°.

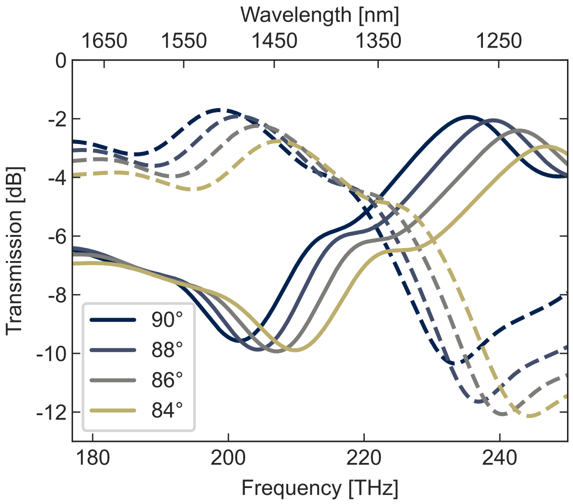

Figure R1: Comparison of an inverse-designed C- and O-band wavelength-division multiplexer simulated with different sidewall angles.

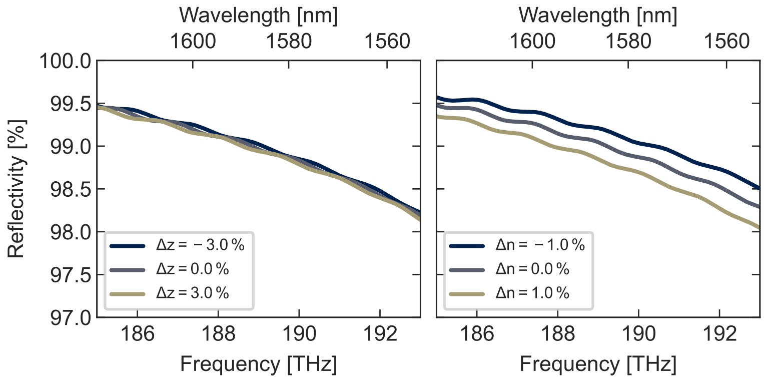

Figure R2: Comparison of an inverse-designed mirror simulated with thickness (left panel) and refractive index (right panel) deviating from the ideal values of $z = 800\,\mathrm{nm}$ and $n = 2$.

Although the optimised structures performance deviates from the design, it can be compensated by sweeping the overall size of the device.

The following text is added to the main text under **Materials and Methods**:

The designs are robust against variations in the film thickness, material index, and sidewall angle as shown in the supplementary information.

While the figures and the subsequent paragraph has been appended to the supplementary information.

The optimisation routine assumes vertical sidewalls, no deviation from the designed thickness, minimum feature sizes achievable during fabrication and constant refractive index. In figures R1 and R2, the impact of fabrication tolerances, such as different thickness, refractive index, and sidewall angle are shown for the inverse-designed mirror and wavelength-division multiplexer shown in this work. In practice, a parametric sweep of the uniform scaling factor is performed to compensate for systematic fabrication deviations.

In Fig. 3d, there is a noticeable reduction in the crosstalk variation and insertion loss compared with that in Fig. 3b. Is this improvement a result of the larger film thickness and optimization

area, or of a different optimization algorithm?

We thank the reviewer for this observation. Indeed, the reason for the variation in performance between Fig. 3b and Fig. 3d arises from the optimization algorithm used for each respective device. In the case of the large-footprint MDM presented in Fig. 3d, the objective function was designed to penalize large variations in both transmission and crosstalk. This choice enforces relatively flat transmission spectra across the entire frequency range, leading to reduced crosstalk variation.

To this effect, we have adjusted the following text to clarify in the **Materials and Methods** section:

For the larger-footprint MDM ($10 \times 15\,\mu m^2$ footprint), a custom inverse-design algorithm was developed using *fdtd-z*, open-source optimization tools, and custom objective functions. This approach enables reduced crosstalk variation and lower insertion loss by penalizing crosstalk variation more heavily. Additional fabrication constraints were also introduced to ensure robust and reproducible fabrication of the large-footprint device.

If my understanding is correct, the only difference between the two F-P cavities in Fig. 4d is the length of the intracavity waveguide. I am curious why the transmission of the TE00 modes in the 307.6 GHz cavity is very different from that in the 144.6 GHz cavity. Did the authors use different reflector designs?

In our work, the same reflector designs were used for both cavities. As the cavity is shortened, the resonance linewidths increase. This means the resonances from different spatial mode families more easily overlap and become harder to distinguish if they have similar resonance frequencies. Many of the resonances of the TE_{00} cannot be distinguished from the resonances of the TE_{10} for the 307.6 GHz cavity.

The resonances around 188 THz illustrate this in a spectral range where it is difficult to separate the TE_{00} mode from the TE_{10} mode in the 307.6 GHz cavity. The cavities were experimentally measured in transmission, through both mirrors, where the transmission of the mirror is frequency-varying. Since the linewidth changes with different cavity lengths, the coupling efficiency from the cavity changes as well. Both these combine to change the measured amplitude of the resonances between different cavity lengths. The following text has been added to the manuscript to clarifying the different transmission:

Both cavities are formed using identical reflectors. The difference in their resonance transmission arises from the balance between intrinsic cavity loss and the reflector outcoupling rates. In addition to this, the shorter cavity has broader linewidths which make distinguishing overlapping modes more difficult. The transmission of both FP cavities in the vicinity of 188 THz is shown in the supplementary information to illustrate this.

While the figure and the accompanying text are added to the supplementary information.

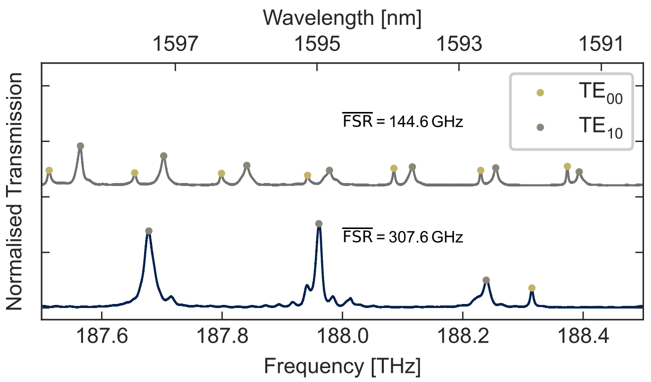

Figure R3: Transmission of inverse-designed FP cavities in the vicinity of 188 THz

As the cavity is shortened, the resonance linewidths increase. This means the resonances from different spatial mode families more easily overlap and become harder to distinguish if they have similar resonance frequencies. Many of the resonances of the TE_{00} mode cannot be distinguished from the resonances of the TE_{10} mode for the 307.6 GHz cavity. The resonances around 188 THz in figure R3 illustrate this in a spectral range where it is difficult to separate the TE_{00} mode from the TE_{10} mode in the 307.6 GHz cavity.

The Q-factor of the F-P cavities is 0.21 million, which seems too low for practical applications. By comparison, a Q-factor of 4 million has been demonstrated in traditionally designed F-P cavities (Ref. 48). I believe the authors should discuss the limiting factors, possible ways to improve the Q-factor of the F-P cavities, and the maximum Q-factor that can be achieved in simulation.

To compare the Q-factors of FP resonators, the cavities should have equivalent lengths or FSRs. The traditionally designed FP cavities in Ref. 48 have an average FSR of around 18.55 GHz within the operation band while our cavities have an average FSR of 144.6 and 307.6 GHz. If we take the same FSR for cavities formed with the mirrors presented, a Q-factor of 1.3 million can be achieved since the cavity losses are dominated by the mirrors rather than the cavity waveguide in-between the mirrors. In the cavities shown here, the single mirror round-trip losses are around 1.5 % while the cavity waveguide losses are around 0.085 % per round-trip for an FSR of 144.6 GHz or 0.045 % per round-trip for an FSR of 307.6 GHz. If the cavity length is increased to 8.5 mm (equivalent to an FSR of 18.55 GHz) the round-trip cavity waveguide losses are still only 0.685 %. Note that each of their reflectors (which include a taper section) are around 400 µm long.

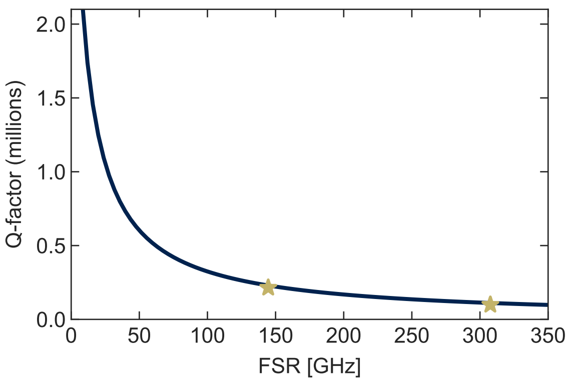

Figure R4: Q-factor of an FP cavity as the FSR is varied, where the markers are the measured cavities in this work.

In the following, the cavities and therefore mirrors demonstrated here are compared to the inverse-designed silicon nitride reflectors previously reported [6]. The mirrors in this work have a peak reflectivity of 98.5 % and a reflectivity above 95 % across a 4 THz bandwidth in a 2D footprint of $2.8 \times 11\,\mu m^2$. While the previously reported inverse-designed reflectors in silicon nitride[6] have a maximum reflectivity of 94.2 ± 2.4 % in a similar 2D footprint of $4 \times 8\,\mu m^2$. This would result in a maximum Q-factor between 46,700 to 118,000 at 144.6 GHz, although a resonator is not reported in their work. In this case, we assume the best-case scenario that the in-cavity waveguide has the same propagation loss as our work. In practice, the single-mode waveguide in that work would have higher propagation losses compared to the multi-mode waveguide used in our work. As well as the higher demonstrated reflectivity, our reflectors are designed to function in transmission further reducing the total required footprint for an accessible FP cavity.

The simulated reflectivity for the quasi-TE_{00} mode is around 99.5 % at 188 THz as shown below.

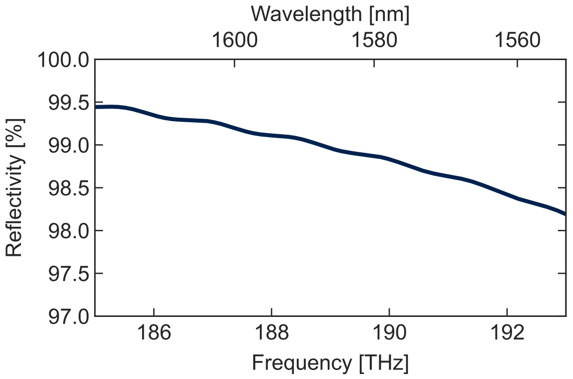

Figure R5: Reflectivity of a single inverse-designed mirror for the quasi-TE_{00} in the same spectral window as shown in this work.

At the end of the **Microresonators** section, we highlight the comparison in the supplementary information with the following:

We compare the FP cavities here to FP cavities formed using 400 µm-long distributed Bragg reflectors [7] and previously reported inverse-designed reflectors on 400 nm-thick SiN [6] in detail in the supplementary information.

Furthermore, the dispersion of the 144.6 GHz FP resonator is compared to a ring resonator fabricated with the same film thickness. The group-velocity dispersion, $D_2/(2\pi)$, of the FP cavity is 86.73 MHz compared to 1.64 MHz from the ring resonator, highlighting the potential for these inverse-designed FP cavities on dispersion engineering (see supplementary information for more details).

Both the comparison to conventional FP cavities and inverse-designed mirrors, and simulated reflectivity for the quasi-TE_{00} mode have been included in the supplementary information along with the following text:

The reflector design determines the microcavity finesse independently of the cavity FSR, and for a given finesse, the cavity Q-factor increases (decreases) as the cavity length increases (decreases). In the cavities shown in this work, the single mirror round-trip losses are 1.5 % while the cavity waveguide losses are 0.08 % per round-trip for an FSR of 144.6 GHz or 0.04 % per round-trip for an FSR of 307.6 GHz. If the cavity length is increased to 8.1 mm (equivalent to an FSR of 18.55 GHz [7]) the round-trip cavity waveguide losses are still only 0.68 %, therefore, the cavities

shown here are mirror-loss limited. At an FSR of 18.55 GHz, the Q-factor is 1.29 million, as shown in figure R4.

The mirrors in this work have a peak reflectivity of 98.5 % and a reflectivity above 95 % across a 4 THz bandwidth in a 2D footprint of $2.8 \times 11\,\mu m^2$. While the previously reported inverse-designed reflectors in SiN [6] have a maximum reflectivity of $94.2 \pm 2.4\,\%$ in a similar 2D footprint of $4 \times 8\,\mu m^2$. This would result in a maximum Q-factor between 46,700 to 118,000 at 144.6 GHz, although a resonator is not reported in their work. In this case, we assume the best-case scenario that the in-cavity waveguide has the same propagation loss as our work. In practice, the single-mode waveguide in that work would have higher propagation losses compared to the multi-mode waveguide used in our work. As well as the higher demonstrated reflectivity, our reflectors are designed to function in transmission further reducing the total required footprint for an accessible FP cavity.

In Fig. 2b and 2d, it would be better to use 'Transmission (dB)' or 'Normalized transmission (dB)' as the label for the vertical axes, and the same applies to Fig. 3b and 3d.

We thank the reviewer for the suggestions and have implemented them in the updated manuscript.

In the second paragraph of Section B, the authors state that "After around 200 iterations the optimized MDM achieves crosstalk below -21 dB and insertion loss of -4 dB..." The frequencies corresponding to these values should be specified, because both the crosstalk and insertion loss vary significantly with frequency in Fig. 3b.

The paragraph is adjusted to account for the frequency dependence as well as to better match the figure caption.

After around 200 iterations the optimised MDM achieves a maximum crosstalk of -18 dB at 197 THz with an insertion loss of -4 dB at 191 THz for a thickness of 400 nm as shown in the opaque curves in Fig. 3b.

Reviewer 2

In this paper, the authors demonstrate a very high Q thick silicon nitride platform and use standard inverse design techniques to demonstrate a 2-mode multiplexer, 2-wavelength multiplexer, and mode selective reflector with unfortunately mediocre insertion loss and overall performance.

The paper also comes at an unfortunate time as a cited preprint [58] demonstrates significantly larger devices with higher performance that perform more complex functions (4 wavelength multiplexer, 4 mode multiplexer, PBS). However, even if this preprint is not considered, the application of standard inverse design techniques to a silicon nitride platform is not unprecedented

(1) Pita Ruiz, Julian L., Frederic Nabki, and Michaël Ménard. "Silicon nitride TM-pass polarizer using inverse design." Optics Express 31.23 (2023): 37892-37899.

(2) Ruiz, Julian L. Pita, Narges Dalvand, and Michaël Ménard. "Inverse-designed 90-degree silicon nitride bends for the C band." Journal of Lightwave Technology (2025).

We thank the reviewer for their time and the detailed comments. We believe that all inverse-designed devices shown in this work are complementary building blocks that advance the densification of photonic integrated circuits, especially for applications related to but not limited to LIDAR, and classical and quantum photonic computing. Since component density can be a bottleneck, we believe it is important to prioritise functionally complete devices rather than maximizing channel count per device. For the WDMs presented here, many relevant system architectures (e.g. telecommunications links using two widely separated bands, dual-band optical coherence tomography, and pump–signal mux/demux stages in integrated amplifiers) only require

pairs of well-separated spectral regions. With an increasing number of integrated platforms introducing gain media (AlOx, SiN, LN, TeOx, etc.) and the ubiquity of co-packaged gain elements, the practical requirement is therefore often for compact, robust two-port WDMs rather than for large multi-port multiplexers.

Additionally, Ref. 58 inverse-designed devices are optimised an order of magnitude more slowly than our devices. Ref. 58 reports that their optimisation takes between 15 minutes to 5.5 hours per iteration step, depending on the device, and up to 450 iterations per device optimisation. We thus infer that the CWDM, MDM, and PBS optimisation of Ref. 58 has taken between 16 days to 94 days, 2 days to 37 days, and 2 days to 17 days, respectively. The lower bound is optimistic as it assumes the shortest time for every iteration step while the pessimistic guess assumes the longest time for each step. Our optimisation methods take between 1 day and 3 days, using weaker and older hardware (4 Nvidia T4 GPUs, 16 GB VRAM each, released in 2018).

In the revised manuscript we also clarify how our inverse-designed reflector relates to previous inverse-designed SiN reflectors. Earlier demonstrations of optimised reflectors are on comparatively thinner SiN cores, whereas our devices operate on a significantly thicker SiN platform (designed to support ultra-high-Q resonators), which imposes different modal, fabrication, and etch-selectivity constraints. We now explicitly compare the reflector footprints and operating conditions, and emphasise that our reflector remains competitive in size while being compatible with this much thicker, low-loss SiN platform and simultaneously enabling the high-Q resonators demonstrated in this work. Changes made to the manuscript comparing SiN reflectors are presented later.

While the high quality factor platform is impressive, as the authors note it is not the first demonstration of this. Moreover, the high quality factor becomes irrelevant when the insertion loss of devices is as high as it is here (exceeding 2dB). In the same vein, the design of a reflector that keeps the field away from the side walls seems completely superfluous as the intrinsic Q is 4 to 20 million and the Q of the demonstrated engineered resonators is 200,000.

We thank the reviewer for the thoughtful comments. While the high-quality factor SiN platforms have been demonstrated previously, the high-quality factor measurements here were demonstrated primarily as a fabrication process benchmark on the same chip to confirm that the platform is not dominated by background propagation losses.

Although the optimisation scheme presented here is common across each of the devices, there are differences between initial simulated conditions. For the device presented in Fig. 2a, b where the insertion losses increase away when away from the centre of the passbands, the modes of interests were optimised using a single frequency at each passband. To improve the insertion losses, we find that it is helpful to include additional frequencies at optimisation constraints within the desired passband. Below we show the comparison between two optimised devices where the only difference number of optimisation constraints.

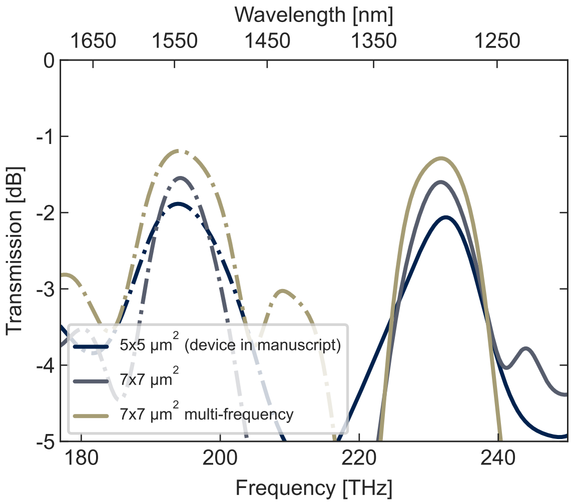

Figure R6: Inverse-designed C- and O-band wavelength-division multiplexer optimised using different initial conditions.

Here, increasing the optimisation area and including additional closely spaced frequencies as objective functions seems to generally improve performance. This is demonstrated by optimising three WDMs with different initial conditions as shown in figure R6. Increasing the optimisation area from $5 \times 5\,\mu m^2$ to an area of $7 \times 7\,\mu m^2$ improved the insertion loss by 0.4 dB in both channels while having two closely spaced in frequency constraints for each channel, separated by 10 nm, instead of one led to 0.8 dB improvement in both channels.

The paragraph below refers to the changes made in the supplementary information.

Increasing the optimisation area and including additional closely spaced frequencies as objective functions seems to generally improve performance. This is demonstrated by optimising three WDMs with different initial conditions as shown in figure R6. Increasing the optimisation area from $5 \times 5\,\mu m^2$ to an area of $7 \times 7\,\mu m^2$ improved the insertion loss by 0.4 dB in both channels while having two closely spaced in frequency constraints for each channel, separated by 10 nm, instead of one led to 0.8 dB improvement in both channels.

The following address the comments on the FP cavities. The reduction of field interaction with the sidewalls by moving away from single-mode waveguides toward multi-mode waveguides is a widely used technique to improve quality factors in ring resonators. However, to suppress unwanted mode couplings, the inverse-designed resonators presented here provide a route toward mode-selective reflection as an additional feature by shifting the reflection bands for the different spatial modes.

To compare the Q-factors of FP resonators, the cavities should have equivalent lengths or FSRs. FP cavities using distributed Bragg reflectors [7] have an average FSR of around 18.55 GHz within the operation band while our cavities have an average FSR of 144.6 GHz and 307.6 GHz. If we take the same FSR for cavities formed with the mirrors presented, a Q-factor of 1.29 million can be achieved since the cavity losses are dominated by the mirrors rather than the cavity waveguide in-between the mirrors. In the cavities shown in this work, the single mirror round-trip losses are 1.5 % while the cavity waveguide losses are 0.08 % per round-trip for an FSR of 144.6 GHz or 0.04 % per round-trip for an FSR of 307.6 GHz. If the cavity length is increased to 8.1 mm (equivalent to an FSR of 18.55 GHz) the round-trip cavity waveguide losses are still only 0.68 %, therefore, the cavities shown here are mirror-loss limited. At an FSR of 18.5 GHz, the Q-factor is 1.29 million, as shown in figure R7.

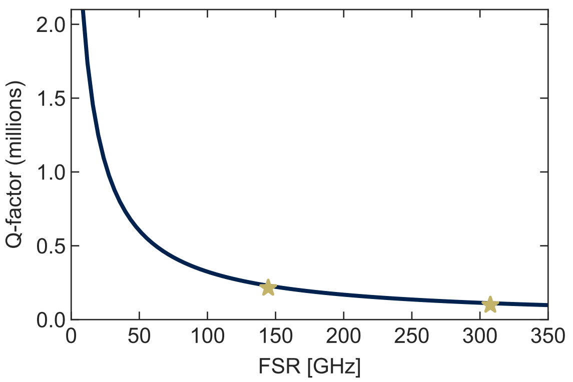

Figure R7: Q-factor of an FP cavity as the FSR is varied, where the markers are the measured cavities in this work.

The mirrors in this work have a peak reflectivity of 98.5 % and a reflectivity above 95 % across a 4 THz bandwidth in a 2D footprint of $2.8 \times 11\,\mu m^2$. While the previously reported inverse-designed reflectors in SiN [6] have a maximum reflectivity of 94.2 ± 2.4 % in a similar 2D footprint of $4 \times 8\,\mu m^2$. This would result in a maximum Q-factor between 46,700 to 118,000 at 144.6 GHz, although a resonator is not reported in their work. In this case, we assume the best-case scenario that the in-cavity waveguide has the same propagation loss as our work. In practice, the single-mode waveguide in that work would have higher propagation losses compared to the multi-mode waveguide used in our work. As well as the higher demonstrated reflectivity, our reflectors are designed to function in transmission further reducing the total required footprint for an accessible FP cavity.

The following paragraph and figures are added to the supplementary information.

The reflector design determines the microcavity finesse independently of the cavity FSR, and for a given finesse, the cavity Q-factor increases (decreases) as the cavity length increases (decreases). In the cavities shown in this work, the single mirror round-trip losses are 1.5 % while the cavity waveguide losses are 0.08 % per round-trip for an FSR of 144.6 GHz or 0.04 % per round-trip for an FSR of 307.6 GHz. If the cavity length is increased to 8.1 mm (equivalent to an FSR of 18.55 GHz [7]) the round-trip cavity waveguide losses are still only 0.68 %, therefore, the cavities shown here are mirror-loss limited. At an FSR of 18.55 GHz, the Q-factor is 1.29 million, as shown in figure R4.

The mirrors in this work have a peak reflectivity of 98.5 % and a reflectivity above 95 % across a 4 THz bandwidth in a 2D footprint of $2.8 \times 11\,\mu m^2$. While the previously reported inverse-designed reflectors in SiN [6] have a maximum reflectivity of 94.2 ± 2.4 % in a similar 2D footprint of $4 \times 8\,\mu m^2$. This would result in a maximum Q-factor between 46,700 to 118,000 at 144.6 GHz, although a resonator is not reported in their work. In this case, we assume the best-case scenario that the in-cavity waveguide has the same propagation loss as our work. In practice, the single-mode waveguide in that work would have higher propagation losses compared to the multi-mode waveguide used in our work. As well as the higher demonstrated reflectivity, our reflectors are designed to function in transmission further reducing the total required footprint for an accessible FP cavity.

Due to the addition of mirrors in our cavity, the flexibility of dispersion with these cavities is much higher than conventional ring resonators which can only adjust the cross-section and/or radius. In this case here (shown in figure R8), the total cavity resonator dispersion is strongly anomalous, $D_2/(2\pi) = 86.73$ MHz, which supports higher power per comb-line in the operational band of the inverse-designed FP cavity. Note that the dispersion noted here is challenging to

reach in a conventional ring geometry, $D_2/(2\pi) = 1.64\,\text{MHz}$ for a ring resonator fabricated on the same chip. In the figure below, the integrated dispersion of a ring resonator is compared to the FP resonator with 144.6 GHz FSR, where the group-velocity dispersion is around 53 times stronger in the FP cavity than the ring resonator. One can envisage the use of the strong GVD in the FP cavity to concentrate more power in the centre of the comb spectrum.

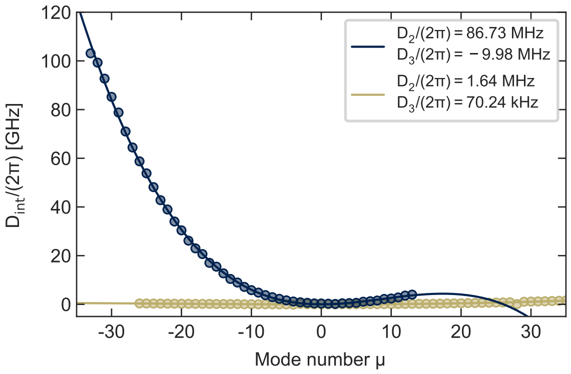

Figure R8: Dispersion of an inverse-designed FP cavity with FSR = 144.6 GHz (blue) compared to a ring resonator on the same chip (gold)

The paragraph and figure above are included in the supplementary information.

Due to the addition of mirrors in our cavity, the flexibility of dispersion with these cavities is much higher than conventional ring resonators which can only adjust the cross-section and/or radius. In this case here (shown in figure R8), the total cavity resonator dispersion is strongly anomalous, $D_2/(2\pi) = 86.73\,\text{MHz}$, which supports higher power per comb-line in the operational band of the inverse-designed FP cavity. Note that the dispersion noted here is challenging to reach in a conventional ring geometry, $D_2/(2\pi) = 1.64\,\text{MHz}$. In the figure below, the integrated dispersion of a ring resonator is compared to the FP resonator with 144.6 GHz FSR, where the GVD is around 53 times stronger in the FP cavity than the ring resonator. One can envisage the use of the strong GVD in the FP cavity to concentrate more power in the centre of the comb spectrum.

Under the section **Microresonators**in the main manuscript, the following text are added to refer to the changes in the supplementary information:

We compare the FP cavities here to FP cavities formed using 400 µm long distributed Bragg reflectors[7] and previously reported inverse-designed reflectors on 400 nm-thick SiN[6] in detail in the supplementary information.

Furthermore, the dispersion of the 144.6 GHz FP resonator is compared to a ring resonator fabricated with the same film thickness. The group-velocity dispersion of the FP cavity is 86.73 MHz compared to 1.64 MHz from the ring resonator, highlighting the potential for these inverse-designed FP cavities on dispersion engineering (see supplementary information for more details).

Additional note: The side wall angle appears to be relatively significant and may explain why some devices perform significantly worse than simulated even when fabrication tolerance is accounted for.

We agree with the reviewer that the sidewall angle – amongst other fabrication variations – can be detrimental in terms of matching the simulated device performance with characterisation of the fabricated devices. Although our optimisations are performed with only minimum feature

sizes as a constraint, it has been shown that incomplete etching leaving a pedestal and sidewall angles can be accounted for in the optimisation routine albeit at a cost of optimisation time [8]. In addition to global process variations, accounting for pattern-dependent variations can be performed with proximity error correction and/or fabrication-aware design [9]. Implementing these algorithmic advancements into our processes would increase the robustness of our method especially as thicker silicon nitride films are required for nonlinear inverse-designed photonics and longer wavelength operation. Additionally, device geometries are routinely parametrised to evaluate fabrication tolerances. We have included simulations that illustrate the impact of fabrication variations on the expected device performance.

In the supplementary information, we have included simulations that show device performance changing with fabrication tolerances such as thickness, refractive index, dispersion, and sidewall angle. The figures below show the change in the inverse-designed mirror reflectivity where the thickness and refractive index are changed from the ideal designed value ($z = 800\,\mathrm{nm}$ and $n = 2$) and the WDM performance as the sidewall angle deviates from 90°

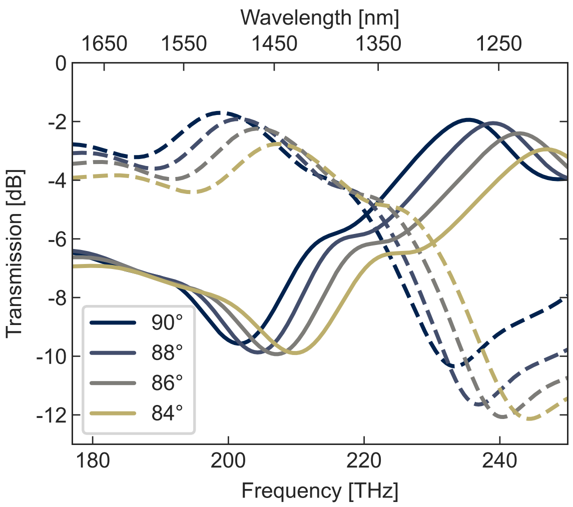

Figure R9: Comparison of an inverse-designed C- and O-band wavelength-division multiplexer simulated with different sidewall angles.

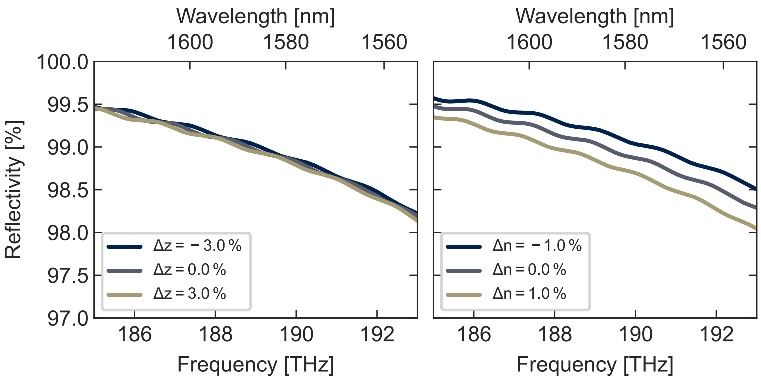

Figure R10: Comparison of an inverse-designed mirror, simulated with thickness (left panel) and refractive index (right panel) deviating from the ideal values of $z = 800\,\mathrm{nm}$ and $n = 2$.

The optimisation routine assumes vertical sidewalls, no deviation from the designed thickness, minimum feature sizes achievable during fabrication and constant refractive index. In figures

R9 and R10, the impact of fabrication tolerances, such as different thickness, refractive index, and sidewall angle are shown for the inverse-designed mirror and wavelength-division multiplexer shown in this work. In practice, a parametric sweep of the uniform scaling factor is performed to compensate for systematic fabrication deviations.

While in the main manuscript, the following text is added under **Materials and Methods**:

The designs are robust against variations in the film thickness, material index, and sidewall angle as shown in the supplementary information.

Although the work is presented well, I cannot recommend its publication in Nature Communications.

We thank the reviewer for their time and the constructive comments that have helped us a lot to clarify our work in the revised manuscript.

References

[1] Ji, X. *et al.* Efficient mass manufacturing of high-density, ultra-low-loss Si_3N_4 photonic integrated circuits. *Optica* **11**, 1397–1407 (2024).

[2] Singh, N. *et al.* Silicon photonics-based high-energy passively Q-switched laser. *Nature Photonics* **18**, 485–491 (2024).

[3] Osornio-Martinez, C. E. *et al.* Monolithically integrated erbium-doped polycrystalline Al_2O_3 waveguide amplifier on silicon photonics platform. *Optics Express* **33**, 23491–23502 (2025).

[4] Shah, P., Mellouk, E. B., Levine, J. & Mohanty, A. Mode-division multiplexing for visible photonic integrated circuits. *Optics Letters* **49**, 5751–5754 (2024).

[5] Luo, L.-W. *et al.* WDM-compatible mode-division multiplexing on a silicon chip. *Nature Communications* **5**, 3069 (2014).

[6] Ruiz, J. L. P., Nabki, F. & Ménard, M. Inverse-designed silicon nitride reflectors. *Optics Letters, Vol. 49, Issue 4, pp. 786-789* (2024).

[7] Wildi, T., Gaafar, M. A., Voumard, T., Ludwig, M. & Herr, T. Dissipative Kerr solitons in integrated Fabry–Perot microresonators. *Optica* **10**, 650–656 (2023).

[8] Kim, C. *et al.* Freeform thin-film lithium niobate mode converter for photon-pair generation. *Nanophotonics* (2025).

[9] Gostimirovic, D., Xu, D.-X., Liboiron-Ladouceur, O. & Grinberg, Y. Deep Learning-Based Prediction of Fabrication-Process-Induced Structural Variations in Nanophotonic Devices. *ACS Photonics* **9**, 2623–2633 (2022).
